# Supplementary material for: Maternal Uniparental Isodisomy of Chromosome 4 and 8 in Patients with Retinal Dystrophy: SRD5A3-Congenital Disorders of Glycosylation and RP1-Related Retinitis Pigmentosa
Source: Genes (Basel). 2022 Feb 16;13(2):359. doi: 10.3390/genes13020359 (PMC8872353; doi:10.3390/genes13020359)
Supplement: Supplementary file 1 [file genes-13-00359-s001.zip › Supplementary Figure S2.pdf]

GTGACACTTTTTTTCCTAGTGATGGTTATGGTGTGGATCAGACTTCTATGAATAAGGCTTGTTTCCTAGGAGAGGTCTGTTCA
CTTACTGATACTGTGTTTTCTGATAAGGCTTGTGCTCAAAAGGAGAACCATACCTATGAGGGAGCTTGCCCAATTGATGAGAC
CTACGTTCCTGTCAATGTCTGCAATACCATTGACTTTTTAAACTCC**AAAGAAAACAC**GGCCGGGCGCGGTGGCTCACGCCTGT
AATCCCAGCACTTTGGGAGGCCGAGGCGGGCGGATCACGAGGTCAGGAGATCGAGACCATCCCGGCTAAAACGGTGAAACCCC
------------------------ undetermined sequences ------------------------------
AAAAAAA**AAAGAAAACAC**ATATACTGATAACTTGGATTCAACTGAAGAGTTAGAAAGAGGTGATGACATTCAGAAAGATCTAA
ATATTTTGACAGACCCTGAATATAAAAATGGATTTAATACATTGGTGTCACATCAAAATGTCAGTAATTTAAGCTCCTGTGGC
CTTTGCCTAAGTGAAAAAGAAGCAGAACTTGATAAGAAACATAGTTCTCTAGATGATTTTGAAAATTGTTCACTA

5′ – *RP1* exon 4 (+ strand) – **Target site duplication** – AluY (+ strand) – **Target site duplication** – *RP1* exon 4 (+ strand) – 3′

Supplementary Figure S2. DNA sequence predicted by *de novo* assembly of the clipped reads. The AluY element insertion was predicted between the duplicated 11 bp segments (target site duplication).
