# Supplementary material for: Maternal Uniparental Isodisomy of Chromosome 4 and 8 in Patients with Retinal Dystrophy: SRD5A3-Congenital Disorders of Glycosylation and RP1-Related Retinitis Pigmentosa
Source: Genes (Basel). 2022 Feb 16;13(2):359. doi: 10.3390/genes13020359 (PMC8872353; doi:10.3390/genes13020359)
Supplement: Supplementary file 1 [file genes-13-00359-s001.zip › Supplementary Table S1.pdf]

Supplementary Table S1. Rare homozygous variants on chromosome 4 in patient 1

Gene	Variant	OMIM (MIM number)	SIFT	PROVEAN	PP2 HVAR	CADD phred	M-CAP
ZNF732	NM_001137608.3:c.1418C>G p.(Pro473Arg)	–	0.017	-7.44	0.921	20.3	-
CFAP99	NM_001193282.4:c.451G>A p.(Asp151Asn)	–	-		-	9.448	-
CPZ	NM_001014447.3:c.1705G>A p.(Ala569Thr)	–	0.628	-0.87	0.012	3.542	0.005
GABRA4	NM_000809.4:c.1051G>A p.(Ala351Thr)	–	0.169	-0.13	0.065	22.5	0.026
NIPAL1	NM_207330.3:c.1019C>A p.(Thr340Asn)	–	0.082	-3.88	0.998	23	0.033
SRD5A3	NM_024592.5:c.57G>C p.(Trp19Cys)	Kahrizi syndrome (612713), Congenital disorder of glycosylation, type Iq (612379)	0.001	-8.02	0.650	26.9	0.290
ENAM	NM_031889.3:c.2529A>C p.(Arg843Ser)	Amelogenesis imperfecta, type IC (204650) Amelogenesis imperfecta, type IB (104500)	0.061	-3.47	0.053	4.748	0.006
ABCG2	NM_004827.3:c.1859A>G p.(Asp620Gly)	–	0.028	-4.97	0.111	22.1	0.060
NR3C2	NM_000901.5:c.218G>A p.(Cys73Tyr)	Pseudohypoaldosteronism type I (177735)	0.031	-1.51	0.002	22.8	0.065
HPGD	NM_000860.6:c.680A>G p.(Asn227Ser)	Hypertrophic osteoarthropathy, primary (259100)	0.238	0	0	13.46	-

-: not available

SIFT (Sorting Intolerant From Tolerant) and PROVEAN (Protein Variation Effect Analyzer)
http://provean.jcvi.org/index.php/ (accessed Sep 3. 2021; GRCh37/Ensembl 66)
SIFT: scores of ≤ 0.05 and those > 0.05 are assessed as damaging and tolerated, respectively.
PROVEAN: scores of ≤ -2.5 and those > -2.5 are assessed as deleterious and neutral, respectively.

Polyphen-2 Hum Var
http://genetics.bwh.harvard.edu/pph2/ (accessed Sep 3. 2021)
HumVar scores were evaluated as 0.000 (most probably benign) to 1.000 (most probably damaging).

CADD (Combined Annotation–Dependent Depletion)
http://cadd.gs.washington.edu/score/ (version 1.6, GRCh38/hg38)
phred scores of > 10–20 are regarded as deleterious, and those of > 20 indicates the 1% most deleterious.

M-CAP (Mendelian Clinically Applicable Pathogenicity) Score
http://bejerano.stanford.edu/mcap/ (accessed Sep 3. 2021; GRCh37/hg19)
scores of ≤ 0.025 and those > 0.025 are assessed as likely benign and possibly pathogenic, respectively.
