# Supplementary material for: Maternal Uniparental Isodisomy of Chromosome 4 and 8 in Patients with Retinal Dystrophy: SRD5A3-Congenital Disorders of Glycosylation and RP1-Related Retinitis Pigmentosa
Source: Genes (Basel). 2022 Feb 16;13(2):359. doi: 10.3390/genes13020359 (PMC8872353; doi:10.3390/genes13020359)
Supplement: Supplementary file 1 [file genes-13-00359-s001.zip › Supplementary Table S2.pdf]

Supplementary Table S2. Rare homozygous variants on chromosome 8 in patient 2

Gene	Variant	OMIM (MIM number)	SIFT	PROVEAN	PP2 HVAR	CADD phred	M-CAP
RP1L1	NM_178857.6:c.6231_6257del p.(Glu2077_Val2085del)	Retinitis pigmentosa 88 (618826) Occult macular dystrophy (613587)	-	-	-	3.967	-
TRMT9B	NM_020844.3:c.806G>C p.(Arg269Thr)	–	0.008	-0.88	0.150	19.28	0.010
RHOBTB2	NM_001160036.2:c.2181C>G p.(Asn727Lys)	Developmental and epileptic encephalopathy 64 (618004)	0.16	-0.82	0.370	16.6	0.019
ADAM18	NM_014237.3:c.494A>G p.(Gln165Arg)	–	0.102	-1.98	0.017	14.98	0.003
DKK4	NM_014420.3:c.199C>T p.(Arg67Cys)	–	0	-6.78	0.078	23	0.067
CDH17	NM_004063.4:c.880G>A p.(Val294Met)	–	0.005	-1.69	0.988	24.8	0.020
MTSS1	NM_001282971.2:c.377A>G p.(His126Arg)	–	0.026	-7.15	0.319	25.4	0.041
OPLAH	NM_017570.5:c.685G>A p.(Val229Ile)	5-oxoprolinase deficiency (260005)	0.008	-0.88	0.996	23.6	0.097

-: not available
For explanation of each score, see footnote of Supplementary Table 1.
