# Supplementary figures and images for: Maternal Uniparental Isodisomy of Chromosome 4 and 8 in Patients with Retinal Dystrophy: SRD5A3-Congenital Disorders of Glycosylation and RP1-Related Retinitis Pigmentosa
Source: Genes (Basel). 2022 Feb 16;13(2):359. doi: 10.3390/genes13020359 (PMC8872353; doi:10.3390/genes13020359)

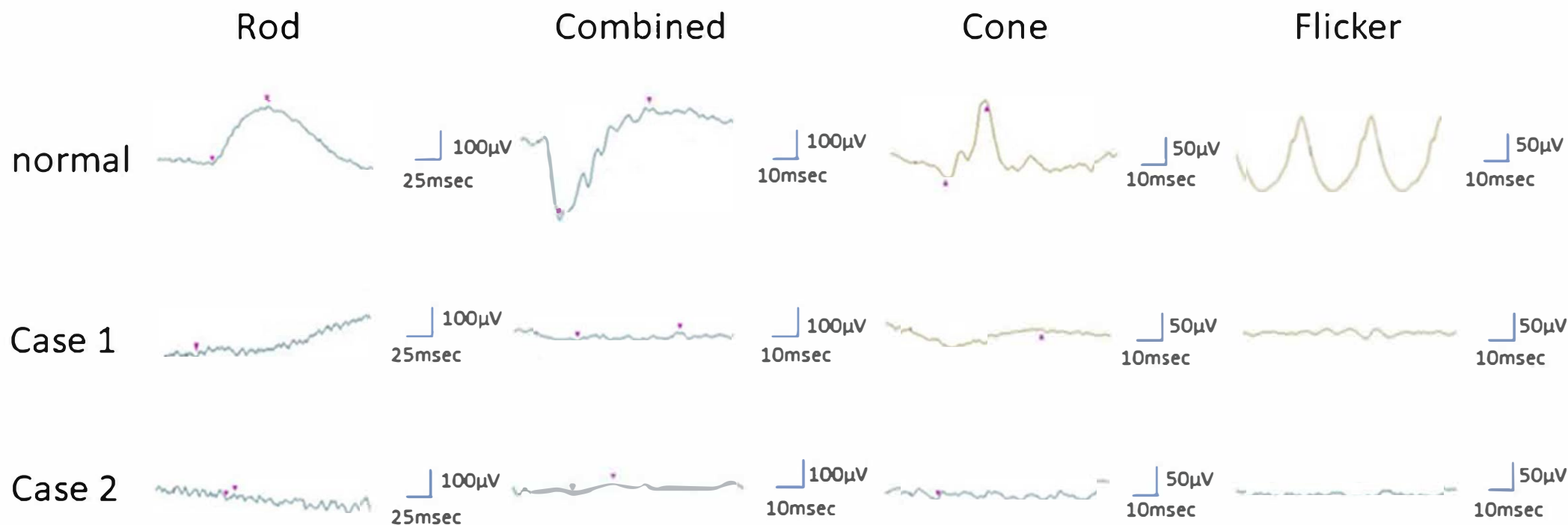

**Supplementary Figure S1.** Full-field electroretinography (ERG).

Supplement: Supplementary file 1 [file genes-13-00359-s001.zip › Supplementary Figure S1.pdf]
